# A Certain Remedy for the Toothache

**Published:** 1831

**Authors:** 


A certain Remedy for the Tooth-ache. By Dr. Ryan.

If the experience of others confirms that of Dr. Ryan, in the simple remedy here proposed for a most distressing malady, he will deserve a civic crown, in addition to the thanks of the profession.

"A remedy which is capable of affording immediate relief to the excruciating pain of tooth-ache, without the slightest pain, being produced by its application, has long been a desideratum; and I feel great gratification in being the medium of proposing such a valuable remedy to the profession.

It is right to observe, that before I resolved upon this course, I deemed it necessary to determine the value of this agent, and to try it upon myself and many other individuals; and ample experience has convinced me of its efficacy.

Like many of our best remedies, that which I proceed to notice was discovered by accident. A gentleman who attends my lectures (Mr. Myers, of

Newington Causeway), had frequently applied sulphuric acid to his tooth with some relief; but on one occasion, he, in a moment of confusion, took down the next bottle to his remedy, which contained nitric acid. To his great surprise, he experienced immediate relief, and without the slightest pain. Since that period he has not suffered from tooth-ache, though three years have now elapsed. During the last winter he informed me of the success of this remedy, which induced me to try it, while labouring under the most intense pain from tooth-ache. The effect was immediate, and no pain whatever was induced. I have since used it in numerous cases, and invariably with complete success. In some instances the disease does not return for days or weeks; and in others not for months.

The best mode of employing it is by means of lint wrapped round a probe, and moistened with the acid, which is then to be slowly applied to the cavity of the tooth; care being taken not to touch the other teeth, the gums or the cheeks. On withdrawing the probe, and inquiring how the patient feels, the usual reply is, 'the pain is entirely gone.' The mouth is next to be washed with tepid water. The acid should be gradually applied to the whole cavity of the tooth, or otherwise a second application will be required before complete relief will be obtained.

This remedy may be used when the gum and cheek are inflamed, so as to preclude the possibility of extraction. In cases where the diseased fang remains, and when the caries faces the adjacent tooth, it obviates the necessity of extraction in all cases of hollow teeth, which all practitioners declare to be desirable, if possible; and it enables the dentist to perform the operation of 'stopping or filling teeth,' much sooner than he can otherwise accomplish. In a word, it will alleviate a vast deal of human suffering, and supersede a most painful operation. It is not a panacea for all the diseases of the teeth and gums, though a certain and efficacious remedy for the most common cause of tooth-ache. It will be a valuable remedy for children, delicate persons, and pregnant women. It does not accelerate the decay of the tooth to which it is applied.

As the employment of this acid in the disease under notice is not recommended in any pharmacopœia, ancient or modern, of these or other countries with which I am acquainted, and as tooth-ache is now a most prevalent complaint, in consequence of the inclemency of the season, I think a more favourable opportunity cannot occur for the communication of the information described in this paper."—*Med. and Surg. Journ.*

